# Emergency Contraception Counseling in California Community Pharmacies: A Mystery Caller Study

**DOI:** 10.3390/pharmacy7020038

**Published:** 2019-04-23

**Authors:** Lindsay Ditmars, Sally Rafie, Gellan Kashou, Kelly Cleland, Lisa Bayer, Tracey A. Wilkinson

**Affiliations:** 1 Department of Pharmacy, UC San Diego Health, San Diego, CA 92103, USA; lditmars@ucsd.edu; 2 Skaggs School of Pharmacy and Pharmaceutical Sciences, University of California San Diego, La Jolla, CA 92093, USA; 3 Birth Control Pharmacist, San Diego, CA 92122, USA; 4 Owl Rexall Drug, Glendora, CA 91740, USA; gkashou18@gmail.com; 5 Office of Population Research, Woodrow Wilson School of Public and International Affairs, Princeton University, Princeton, NJ 08540, USA; kcleland@princeton.edu; 6 Department of Obstetrics and Gynecology, Oregon Health and Science University, Portland, OR 97239, USA; bayerl@ohsu.edu; 7 Children’s Health Services Research, Department of Pediatrics, Indiana University School of Medicine, Indianapolis, IN 46202, USA; tracwilk@iu.edu

**Keywords:** emergency contraception, levonorgestrel, ulipristal acetate, pharmacy, pharmacist

## Abstract

This study was conducted to determine which emergency contraception (EC) methods are offered by community pharmacists in response to patient calls. Female mystery callers called all community pharmacies in two California cities using standardized scripts. The callers inquired about options available to prevent pregnancy after sex and whether that method was available at the pharmacy, using follow-up probes if necessary. A total of 239 calls were completed in San Diego (*n* = 127, 53%) and San Francisco (*n* = 112, 47%). Pharmacists indicated availability at most sites (*n* = 220, 92%) with option(s) reported as levonorgestrel only (LNG; *n* = 211, 88.3%), both ulipristal acetate (UPA) and LNG (*n* = 4, 1.6%), UPA only (*n* = 1, 0.4%), or non-specific EC (*n* = 4, 1.7%). Nineteen pharmacies (7.9%) did not have EC available on the day of the call. Following additional probing, some pharmacists discussed UPA (*n* = 49, 20.5%) or the copper intrauterine device (*n* = 1, 0.4%) as EC options. LNG EC products were available same-day in 90.1% of pharmacies, whereas UPA was available same-day in 9.6% of pharmacies. The majority of pharmacies called in this study offered and stocked at least one EC option, but the focus of discussions was on LNG and matched what was in stock and available.

## 1. Introduction

Emergency contraception (EC) is a safe method of contraception that can be used to prevent pregnancy after unprotected intercourse, including contraceptive failure. There are three forms of EC currently available in the United States: the copper intrauterine device (Cu-IUD), oral ulipristal acetate (UPA), and oral levonorgestrel (LNG) [1]. LNG 1.5 mg single tablet (sold as Plan B-One Step^®^ and many generic products) continues to be the most widely used EC method due to its time on the market (since 1999) and over-the-counter availability (with age and point-of-sale restrictions from 2006 to 2013 and without any restriction in 2013) [2]. UPA 30 mg single tablet (sold as ella^®^) was approved for use in the United States in 2010 and is available by prescription only, though adoption into clinical practice has been slow [3].

There are several barriers to access for EC, including the prescription requirement for UPA and need for a trained clinician to place the Cu-IUD. Healthcare provider knowledge about EC is another ongoing barrier to access. One recent study showed that only 29% of healthcare providers have heard of UPA EC and only 7% provide UPA EC [1]. However, both UPA and the Cu-IUD are more effective than LNG at preventing pregnancy up to five days after unprotected intercourse [4,5,6]. Research suggests that LNG may be less effective in women with higher weight or body mass index [7]. To further complicate appropriate product selection, there are theoretical drug interaction concerns with routine hormonal contraceptives and UPA [8].

Pharmacists in California and other states that allow pharmacist prescribing of EC stand in the particular and critical position of being able to provide both prescription and over-the-counter access to oral EC, as well as counseling on all EC methods. With both the over-the-counter availability of LNG and a California Board of Pharmacy protocol allowing trained pharmacists to prescribe oral EC after completing one-hour of continuing education specific to EC to be able to participate in the protocol, there is an ever-increasing opportunity to expand access to EC through pharmacists and pharmacies [4,9].

The primary objective of this study is to determine which EC methods pharmacists in community pharmacies offer and discuss with an inquiring caller. Secondary objectives included evaluating whether any pharmacy characteristics are associated with more comprehensive counseling and to assess the availability of UPA in community pharmacies. 

## 2. Materials and Methods

**Figure 1:**
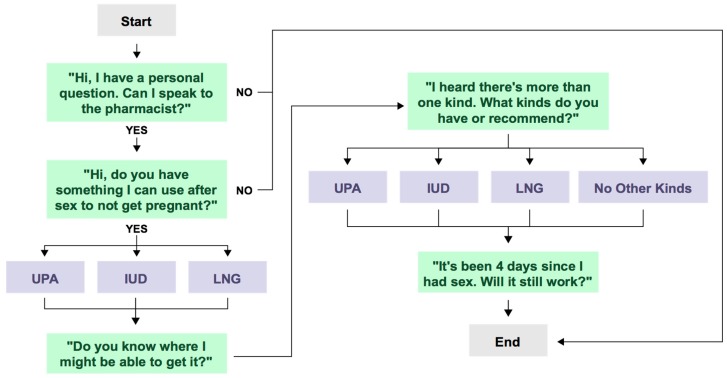
Call script.

The study team identified all community pharmacies in the cities of San Diego and San Francisco (defined by city listed in the full postal address of the pharmacy) with an active license using the California Board of Pharmacy public website list of licensed pharmacies. Pharmacies were randomized and assigned equally between two investigators (GK and LD) to complete calls from August to November 2016. The researchers posed as adult 21-year-old females calling the pharmacy to inquire about pregnancy prevention options after having unprotected sex. Callers followed a structured script (Figure 1) to assess same day availability of all EC products presented by the pharmacist. 

The callers collected data during the calls using an online data collection tool (Google Forms) with restricted access. Pharmacies were described as either chain (four or more locations), grocery store, big box (also known as mass merchandiser) or independent. 

Calls were made between 10:00 a.m. and 8:00 p.m. Monday through Saturday. If a pharmacist was unavailable or the phone was not answered, researchers made two follow up calls between 10:00 a.m. and 5:00 p.m. Monday through Friday to ensure business hours for a total of three attempts. Phone calls took between 30 s and 20 min, and if the call was placed on hold for 20 min or the pharmacist was not available, the call was ended and determined to be an unsuccessful attempt. The call was determined to be completed if, after asking for a pharmacist, the call was then taken by a pharmacist. 

The categorical data was analyzed by Pearson Chi squared tests using STATA MP software version 15.1 (College Station, TX, USA). Significance was set at *p* <0.05. The University of California San Diego Human Research Protections Program institutional review board approved the study protocol.

## 3. Results

The study team analyzed completed calls (*n* = 239, 98.4% of calls made). The remaining calls were not completed because the pharmacist was not available or refused to take the call. Only 18 (7.5%) pharmacies required more than one attempt to reach a pharmacist.

Approximately half of the pharmacies were in each city, with 53.1% in San Diego (127/239) and 46.9% in San Francisco (112/239). Of the 239 calls, 61.5% (147/239) were chain pharmacies, 17.6% (42/239) were independent pharmacies, 13.4% (32/239) were grocery store pharmacies and 7.6% (18/239) were big box pharmacies. 

After being asked about options for pregnancy prevention after unprotected sex, most pharmacists presented LNG alone (88.3%, 211/239), while few pharmacists mentioned both UPA and LNG (1.7%, 4/239) or UPA alone (0.4%, 1/239). The remaining 9.6% (23/239) did not mention EC methods by name or did not have EC. After the callers asked additional probing questions regarding options for EC, specifically, “I heard there’s more than one kind. What kinds do you have or recommend?” and mentioned that unprotected sex had occurred four days prior, the most pharmacists, 20.5% (49/239) then mentioned UPA, 0.4% (1/239) then mentioned LNG, and 0.4% (1/239) then mentioned Cu-IUD (Table 1). Among the pharmacists who mentioned LNG during the call, 99.1% (214/216) had it in stock; in comparison, only 43.4% (23/53) of the pharmacists who offered UPA during the call had UPA in stock (Table 2). LNG EC products were available same-day in 90.1% of pharmacies, whereas UPA was available same-day in 9.6% of pharmacies.

There was no statistically significant difference in whether UPA was discussed or available by city or by pharmacy type. Discussion and stocking of LNG did not vary by city. Pharmacists at chain stores were more likely to mention and stock LNG EC (*p* < 0.001). 

The pharmacists answered all of the caller’s questions in 82.8% (198/239) of the completed calls. Most of the pharmacists (88.7%, 212/239) mentioned the timeframe for taking the recommended product at some point throughout the call. Most pharmacists did not ask details regarding when the unprotected intercourse took place (74.0%, 177/239), age of the caller (90.7%, 217/239), or weight of the caller (92.5%, 221/239). No pharmacists asked about sexually transmitted diseases (STDs) or counseled about STD screening, treatment, or referral. Few pharmacists (2.1%, 5/239) counseled callers regarding how to continue with ongoing hormonal contraception. Six pharmacists (2.5%) asked about health or prescription insurance. Thirty-nine (16.3%) pharmacists asked about prescriptions (for example asking if the caller needed a prescription or had a prescription) for hormonal contraception or emergency contraception.

## 4. Discussion

During the mystery calls, an overwhelming majority of pharmacists mentioned only LNG for EC, when asked about what to use after unprotected sex to prevent pregnancy. This did not differ between the two California cities in this study. However, more pharmacists from chain pharmacies mentioned LNG as a specific form of EC at some point during the phone conversation than independent pharmacies. Chain pharmacies were also more likely to stock LNG EC than independent pharmacies. It is unknown why this differed by pharmacy type and further study is warranted.

Although both UPA and the Cu-IUD are more effective methods of EC, we found that the large majority of pharmacists did not mention these methods even when prompted that unprotected intercourse occurred four days ago. Additionally, more pharmacies routinely stocked LNG but not UPA. This did not differ between cities. 

Our study results are similar to other studies examining availability of LNG and UPA EC. Prior studies in individual or multiple states found UPA available in 3–10% of pharmacies [10,11,12]. Availability of LNG EC in pharmacies across the U.S. has been demonstrated in many studies over the years. Due to the many change to LNG EC access over the years, the most relevant studies are those in the last few years that found 80–83% same-day availability of LNG EC, compared to 90% in this study [13,14,15]. 

In conducting this study, several limitations were encountered. First, although we called all community pharmacies in two cities, these results may not be applicable to the entire state or less urban areas. Second, this study was conducted in California, a state where pharmacists have prescriptive authority under statewide protocol for all forms of oral EC (since 2004), as well as self-administered hormonal contraceptives (since 2016), following training. For this reason, California pharmacists may have obtained additional knowledge and counseling skills regarding EC. Thus, our findings are likely different from other states where pharmacists do not have prescriptive authority for EC. Third, callers did not ask if LNG and UPA were available in the pharmacy until the method was mentioned by the pharmacist. Therefore, it is possible that a method may have been available at a pharmacy, but if the pharmacist did not mention it, we would not have captured availability. Although we may not have captured true stocking EC at all of the pharmacies called, our approach more closely reflects the real-world experience of patients calling about EC. Results may have varied if the interaction had occurred in person at the pharmacy rather than by phone as well. Lastly, our study assessed for the mention of the Cu-IUD although this cannot be provided or placed in the pharmacy. Our results do not reflect pharmacist knowledge about the Cu-IUD because we did not probe for this. 

Despite limitations, this study was designed to capture the patient’s experience when calling the pharmacy seeking EC. While most pharmacists offered the patient at least one option that was available at the pharmacy that day, a small minority did not offer any EC and most did not offer the most appropriate oral EC given the patient’s circumstance given days since unprotected intercourse. Patients likely look to their community pharmacy as the primary source of EC when needed and timely information and access to the most appropriate product critical for effectiveness. Additional interventions are needed to increase availability of EC in pharmacies, particularly UPA. We suggest interventions such as education in the pharmacy curricula, as well as academic detailing and continuing education programs for practicing pharmacists. Other pharmacy staff besides the pharmacist could benefit from educational interventions as well to enhance the patient experience since other staff often interface with patients first. 

To our knowledge, our study is the first to evaluate EC methods offered by community pharmacists in California since the availability of UPA. Future studies are needed to determine why pharmacists are more likely to discuss LNG with patients than UPA. This could be due to the fact that LNG has been on the market longer than UPA and is available OTC; however other factors may exist and should be investigated further. This study demonstrates gaps in information, services and products that are available to patients at the time of need. Additional research conducted directly with pharmacists around their knowledge, attitudes, practices, and implementation of prescribing can build upon these findings.

## 5. Conclusions

Our study demonstrates some disparities between available forms of EC and those discussed and stocked by pharmacists in community pharmacies. Pharmacists should be offered educational opportunities to improve their knowledge about EC options and counseling skills. A patient-centered counseling approach regarding could greatly benefit patients and increase use of the most effective forms of EC. In order for counseling to lead to increased use and access, pharmacies must also increase same-day availability of UPA.

## Figures and Tables

**Table 1:** Emergency contraception (EC) options discussed (*n* = 239).

Method(s) Discussed Initially	*n* (%)	Additional Method(s) Discussed after Probing	*n* (%)
Levonorgestrel and Ulipristal acetate	4 (1.7%)	Levonorgestrel	–
		Ulipristal acetate	–
		Copper IUD	–
		Unspecified	–
		None	4 (1.7%)
Levonorgestrel	211 (88.3%)	Levonorgestrel and Ulipristal acetate	–
		Ulipristal acetate	48 (20.1%)
		Copper IUD	1 (0.4%)
		Unspecified	–
		None	162 (67.8%)
Ulipristal acetate	1 (0.4%)	Levonorgestrel and Ulipristal acetate	–
		Levonorgestrel	–
		Copper IUD	–
		Unspecified	–
		None	1 (0.4%)
Copper IUD *	0	Levonorgestrel and Ulipristal acetate	–
		Levonorgestrel	–
		Ulipristal acetate	–
		Unspecified	–
		None	–
Unspecified **	4 (1.7%)	Levonorgestrel and Ulipristal acetate	1 (0.4%)
		Levonorgestrel	1 (0.4%)
		Ulipristal acetate	–
		Copper IUD	–
		None	2 (0.8%)
None ***	19 (7.9%)	Levonorgestrel and Ulipristal acetate	–
		Levonorgestrel	–
		Ulipristal acetate	–
		Copper IUD	–
		Unspecified	–
		None	19 (7.9%)

* IUD = intrauterine device; ** Did not mention any specific EC method when answering question: “Hi, do you have something I can use after sex to not get pregnant?”; only responding with Yes; *** Either pharmacist did not mention an EC method (*n* = 4) or they did not carry any EC at their pharmacy (*n* = 15).

**Table 2:** Emergency contraception methods presented and availability by city and pharmacy type.

	San Diego	San Francisco	*p*-Value	Chain	Independent	*p*-Value
	(*n* = 127)	(*n* = 112)		(*n* = 197)	(*n* = 42)	
LNG Discussed	113 (89%)	103 (92%)	*p* = 0.434	191 (96.9%)	25 (59.5%)	*p* < 0.001 *
UPA Discussed	24 (18.9%)	30 (26.8%)	*p* = 0.146	47 (23.9%)	7 (16.7%)	*p* = 0.312
LNG Available	111 (87.4%)	103 (92%)	*p* = 0.250	190 (96.4%)	24 (57.1%)	*p* < 0.001 *
UPA Available	10 (7.9%)	13 (11.6%)	*p* = 0.329	22 (11.1%)	1 (2.4%)	*p* = 0.080

* *p* < 0.05 statistically significant.

## References

[1] Batur P., Cleland K., McNamara M., Wu J., Pickle S., EC Survey Group (2015). Emergency contraception: A multispecialty survey of clinician knowledge and practices. Contraception.

[2] American College of Obstetricians and Gynecologists (2012). Committee Opinion No. 542: Access to emergency contraception. Obstet. Gynecol..

[3] Rafie S., Stone R.H., Wilkinson T.A., Borgelt L.M., El-Ibiary S.Y., Ragland D. (2017). Role of the community pharmacist in emergency contraception counseling and delivery in the United States: Current trends and future prospects. Integr. Pharm. Res. Pract..

[4] Lalitkumar P., Berger C., Gemzell-Danielsson K. (2013). Emergency contraception. Best Pract. Res. Clin. Endocrinol. Metab..

[5] Cleland K., Zhu H., Goldstuck N., Cheng L., Trussell J. (2012). The efficacy of intrauterine devices for emergency contraception: A systematic review of 35 years of experience. Hum. Reprod..

[6] Glasier A.F., Cameron S.T., Fine P.M., Logan S.J., Casale W., Van Horn J., Sogor L., Blithe D.L., Scherrer B., Mathe H. (2010). Ulipristal acetate versus levonorgestrel for emergency contraception: A randomised non-inferiority trial and meta-analysis. Lancet.

[7] Kapp N., Abitbol J., Mathé H., Scherrer B., Guillard H., Gainer E., Ulmann A. (2014). Effect of body weight and BMI on the efficacy of levonorgestrel emergency contraception. Contraception.

[8] American Society for Emergency Contraception (2016). Providing Ongoing Hormonal Contraception after Use of Emergency Contraceptive Pills. http://americansocietyforec.org/uploads/3/4/5/6/34568220/asec_fact_sheet-_hormonal_contraception_after_ec.pdf.

[9] California Board of Pharmacy Emergency CONTRACEPTION Protocol. https://www.pharmacy.ca.gov/publications/ec_protocol.pdf.

[10] Brant A., White K., St Marie P. (2014). Pharmacy availability of ulipristal acetate emergency contraception: An audit study. Contraception.

[11] Bullock H., Steele S., Kurata N., Tschann M., Elia J., Kaneshiro B., Salcedo J. (2016). Pharmacy access to ulipristal acetate in Hawaii: Is a prescription enough?. Contraception.

[12] Shigesato M., Elia J., Tschann M., Bullock H., Hurwitz E., Wu Y.Y., Salcedo J. (2018). Pharmacy access to ulipristal acetate in major cities throughout the United States. Contraception.

[13] Wilkinson T.A., Clark P., Rafie S., Carroll A.E., Miller E. (2017). Access to emergency contraception after removal of age restrictions. Pediatrics.

[14] Wilkinson T.A., Rafie S., Clark P.D., Carroll A.E., Miller E. (2018). Evaluating community pharmacy responses about levonorgestrel emergency contraception by mystery caller characteristics. J. Adolesc. Health.

[15] Uysal J., Tavrow P., Hsu R., Alterman A. (2019). Availability and accessibility of emergency contraception to adolescent callers in pharmacies in four Southwestern states. J. Adolesc. Health.

